# The pathogenicity of T cell epitopes on human Goodpasture antigen and its critical amino acid motif

**DOI:** 10.1111/jcmm.13134

**Published:** 2017-03-10

**Authors:** Shui‐yi Hu, Qiu‐hua Gu, Jia Wang, Miao Wang, Xiao‐yu Jia, Zhao Cui, Ming‐hui Zhao

**Affiliations:** ^1^ Renal Division Peking University First Hospital Beijing China; ^2^ Institute of Nephrology Peking University Beijing China; ^3^ Key Laboratory of Renal Disease Ministry of Health of China Beijing China; ^4^ Key Laboratory of CKD Prevention and Treatment Ministry of Education of China Beijing China; ^5^ Department of Nephrology Tianjin Medical University General Hospital Tianjin China; ^6^ Peking‐Tsinghua Center for Life Sciences Beijing China

**Keywords:** anti‐GBM disease, Goodpasture antigen, epitope, T cell, experimental autoimmune glomerulonephritis

## Abstract

Goodpasture antigen, the non‐collagenous domain of α3 chain of type IV collagen [α3(IV)NC1], is the target antigen of anti‐glomerular basement membrane (GBM) antibodies. The pathogenicity of T cell epitopes is not elucidated clearly. In this study, we aim to define the nephritogenic T cell epitopes and its critical amino acid residues. Twenty‐four overlapping linear peptides were synthesized covering the whole sequence of human α3(IV)NC1. Wistar–Kyoto rats were immunized with linear peptides, and experimental autoimmune glomerulonephritis was evaluated. Critical amino acid was identified by the loss of nephritogenic function after each amino acid substitution by alanine. Of the 24 peptides, P14 (α3_127‐148_) could induce 90.5% (19/21) of WKY rats developing anti‐GBM glomerulonephritis with proteinuria, elevated serum urea and creatinine, IgG linear deposit on GBM and substantial (in average 82.4 ± 5.6%) crescent formation in glomeruli. Lymphocytes of immunized rats proliferated in response to α3_127‐148_ and α3(IV)NC1 *in vitro*. Sera of these rats recognized α3_127‐148_ and later on together with intact human α3(IV)NC1. Antibodies towards α3_127‐148_ and intact α3(IV)NC1 could also be detected from the kidney elutes. These antibodies showed no cross‐reaction with each other, which implies intramolecular epitope spreading during disease progress. After sequential amino acid substitution, the α3_127‐148_ with substitution of tryptophan_136_, isoleucine_137_, leucine_139_ or tryptophan_140_ lost its nephritogenicity. Human α3_127‐148_ is a nephritogenic T cell epitope in WKY rats, with the critical amino acids as W_136_I_137_xL_139_W_140_. These findings might facilitate future investigation on microbial aetiology and potential specific immunotherapy of anti‐GBM disease.

## Introduction

Goodpasture disease, also known as anti‐GBM disease, is an autoimmune disorder characterized by the production of anti‐GBM autoantibodies, rapidly progressive glomerulonephritis and high risks for alveolar haemorrhage. The autoantigen of anti‐GBM disease has been identified as the non‐collagenous domain 1 (NC1) of α3 chain of type IV collagen (α3(IV)NC1), also known as Goodpasture antigen 1, 2. Two major immunodominant regions, E_A_ and E_B_, have been mapped to the residues 17‐31 and 127‐141 of α3(IV)NC1 3, 4 (all amino acid numbering, including other cited epitopes, converted from the original numbering to that of reference 2).

The pathogenic role of anti‐GBM antibodies has been demonstrated in passive transfer studies 5, 6, 7, 8, 9. In recent years, compelling studies indicate that T lymphocytes play an important and independent role on disease pathogenesis in both human Goodpasture disease and animal models. In patients with anti‐GBM disease, T cell infiltration had been found around the affected glomeruli and correlated with the severity of kidney injury 10, 11. T cell involvement can be implied from the strong HLA associations with disease 12, 13, 14, 15. Help from T cells is required for antibody response like affinity maturation, subclass switching, antigen specificity and epitope spreading 16, 17, 18, 19, 20. The animal models provide more direct evidence that T cells participated in the pathogenesis of crescentic nephritis. In the absence of antibodies against GBM, α3(IV)NC1‐specific CD4^+^ T cells alone are sufficient to initiate glomerular injury in WKY rats 21, 22, 23, 24. The disease can be inhibited by anti‐T cell therapies, including CD28–B7 or CD154–CD40 costimulatory blockade 25, 26, and anti‐CD4 and anti‐CD8 monoclonal antibodies 27. Furthermore, T cell tolerance can be induced *via* oral or nasal administration of Goodpasture antigen prior to induction of disease 28, 29.

One study on T cell epitope mapping identified two major T cell epitopes in patients with this disease, as α3_69‐88_ and α3_129‐148_ 30. Our recent findings confirmed the peptide α3_129‐148_ as the major T cell epitope and revealed another T cell epitope as α3_189‐208_ [Hu SY, *et al*. Submitted]. Whether these epitopes are pathogenic to induce glomerulonephritis remains unknown. Using HLA‐DRB1 transgenic mice, Ooi *et al*. successfully defined a HLA‐DRB1*15:01‐restricted T cell epitope, α3_135‐145_, which could induce T cell responses and injury in anti‐GBM nephritis. Four critical amino acid residues for T cell responses were further identified as isolucine_137_, tryptophan_140_, glycine_142_ and phenylalanine_143_ in the corresponding human α3 sequences 31.

In this study, we synthesized a set of peptides spanning the entire sequence of human α3(IV)NC1. The nephritogenic role of one T cell peptide on human Goodpasture antigen was identified by its capacity of inducing crescentic glomerulonephritis in Wistar–Kyoto (WKY) rats. Epitope spreading of B cell reactivity evolved during disease development, which was comparable to what happened in patients with anti‐GBM disease. Critical amino acid residues of this nephritogenic T cell epitope were further identified by sequential amino acid substitution. These findings are advantageous to understand the pathogenesis and aetiology of human Goodpasture disease and might be useful for the potential development of more specific immunotherapies.

## Materials and methods

### Preparation of antigens

#### Linear peptides

The published sequence of human α3(IV)NC1 was used to synthesize peptides 30. A panel of 24 peptides was synthesized covering the whole sequence of human α3(IV)NC1, based on a series of 20‐mer peptides overlapping with 10 amino acids. P14 was truncated into three 13‐mer peptides overlapping by eight amino acids to identify the core immunogenic region of P14. To identify the critical amino acid, each amino acid residue of P14 (α3_127‐148_) was replaced by alanine in a sequential order. All peptides were synthesized on an automatic peptide synthesizer using F‐moc (9‐fluorenyl‐methyloxycarbonyl) chemistry (Beijing Scilight Biotechnology Ltd Co, Beijing, China) and purified by a reverse‐phase CIS column on a preparative HPLC. Purified peptides were analysed by HPLC for purity and mass spectrometry for the correct sequence. Peptides with purity >98% were used for further tests.

#### Recombinant human α(IV)NC1

The recombinant proteins were produced as described previously 19. In brief, cDNA from the NC1 domain of human type IV collagen α1, α2, α3, α4 and α5 was ligated to a type X collagen triple‐helix leader sequence and subcloned into the pcDNA3 vector. The constructs were then stably transfected into a human embryonic kidney cell line (HEK 293), and recombinant proteins were harvested and purified from the medium.

#### Bovine α(IV)NC1

Bovine α(IV)NC1 was prepared as described previously 17. In brief, glomeruli were isolated by differential sieving, and cells were lysed in 5 mmol/l Tris‐HCl at 4°C overnight. Crude GBM was suspended in 4% (w/v) deoxycholic acid at 37°C for 2.5 hrs, in deoxyribonuclease I (50Kunitz U/ml) at 37°C for 1 hr and then digested by bacterial collagenase I (Sigma‐Aldrich, St. Louis, MO, USA) at 1:10 enzyme/protein ratio, in N‐hydroxyethylpiperazine‐N’‐ethane sulphonic acid buffer at 37°C for 20 hrs. The supernatant (soluble GBM proteins) was applied to a Resource Q ion exchange chromatography column (Amersham Pharmacia, Freiburg, Sweden). NC1 domains, which did not bind to the column, were collected, concentrated and dialysed.

### Cells proliferation assays

Lymphocytes isolated from draining lymph nodes of rats 2 weeks after immunization were cultured at a concentration of 5 × 10^5^ cells/well in RPMI 1640 media with 10% FBS, 2 mmol/l L‐glutamine, 100 U/ml penicillin and 0.1 mg/ml streptomycin (ScienCell, Sandiego, CA, USA). The cell proliferation was estimated 72 hrs after stimulation with P14 at 10 μg/ml or human α3(IV)NC1 at 10 μg/ml.

All plates were incubated at 37°C in a humidified atmosphere of 5% CO2/95% air. ^3^H‐thymidine was added to the triplicate microtitre wells at 1 μCi/well, and 16 hrs later, the cells were harvested on an automatic cell harvester (Tomtec, San Diego, CA, USA). Thymidine incorporation was measured using an automated β‐counter (EG&G Wallac, Turku, Finland). The results were expressed in stimulation index (SI) (^3^H‐TdR incorporation [cpm] of cells with antigen stimulation/^3^H‐TdR incorporation [cpm] of cells without antigen stimulation). SI > 2.0 was interpreted as significant positive response 32, 33.

### EAG models

#### Immunization on WKY rats

Female WKY rats, 4–6 weeks of age, were purchased from Vital River Laboratories (Beijing, China). Rats were immunized with either 1.4 μg/g of bovine α(IV)NC1 or 0.7pmol/g of peptides, both emulsified in complete Freund's adjuvant (CFA; Sigma‐Aldrich), by single intraperitoneal injection. Negative controls were immunized with CFA alone. Twenty‐four‐hour urine samples were collected before and after immunization at each week using metabolism cages. Blood samples were collected before and after immunization at each week by angular venipuncture. The experimental animals were killed around 7 weeks after immunization, and blood and kidneys were collected for further experiments. All animal experiments were approved by the Experimental Animal Ethics Committee of Peking University First Hospital (Beijing, China).

#### Evaluation of clinical manifestations and renal histopathology

The urinary protein, serum creatinine and blood urea nitrogen (BUN) of rats were tested in Clinical Laboratory of Peking University First Hospital. Kidney tissues of rats were fixed in 10% buffered formalin, dehydrated through graded ethanol and embedded in paraffin, and kidney sections (3 μm thick) were stained with periodic acid–Schiff or HE staining. At least 50 glomeruli from each rat were observed to assess lesions including glomerular necrosis and crescent formation. Glomerular crescent was defined as at least two layers of cells in Bowman's space 31. The interstitium lesions were scored from 0 to 3+, including inflammatory cell infiltration, tubular atrophy and fibrosis. For electronic microscopy, kidney tissues was fixed in 3% glutaraldehyde and then 1% osmium tetroxide, followed by dehydration in graded ethanol and washing in acetone, and finally embedding in Epon 812. Ultrathin sections were stained with uranyl acetate and lead citrate and examined with a transmission electron microscope (JEM‐1230, JEOL, Japan). For direct immunofluorescence, the 5‐μm frozen sections was fixed in acetone for 10 min. at 4°C, blocked with 3% BSA and stained with FITC‐conjugated goat anti‐rat IgG (Zhongshan Golden Bridge Biotechnology, Beijing, China).

#### Elution of antibodies from kidney

Kidneys were eluted by glycine method as previously described 34. In brief, the cortical portions of the kidneys were dissected out by slicing with a scalpel blade, mixed with cold PBS and homogenized with homogenizer. This homogenate was spun at 2500 ***g*** for 10 min. at 4°C. The sediment was washed with cold PBS five times by centrifugation. After the final wash, the sediment was suspended in five times volume of 0.1 mol/l glycine, PH 2.8 and incubated at 4°C with constant shaking for 2 hrs. After incubation, the mixture was spun at 10,000 ***g*** for 30 min. at 4°C. The supernatant was removed, immediately brought to pH 7.0 with 2 mol/l Tris‐HCl (PH 9.0) and dialysed against several changes of PBS.

### Antibody detections

The antigen spectrum of circulating antibodies and kidney elutes was investigated by ELISA as previous reports 35. Peptides at 10 μmol/l and human α(IV)NC1 at 2 mg/l were diluted with 0.05 mol/l bicarbonate buffer (pH 9.6) and coated onto half of the wells of a polystyrene microtitre plate (Nunc, Roskild, Denmark). The other half of the wells was coated with bicarbonate buffer alone as antigen‐free wells to exclude non‐specific binding. The volume of each well was 50 μl. Incubation was carried out at 4°C overnight. Test sera were diluted at 1:100, and elutes were diluted at 1:5 in PBS containing 0.05% Tween‐20 and added in duplication to both antigen‐coated wells and antigen‐free wells at 37°C for 30 min. After washing thrice, alkaline phosphatase‐conjugated goat anti‐rat IgG (Sigma‐Aldrich) diluted 1:4000 was added. Incubation resumed at 37°C for 30 min. After washing, p‐nitrophenyl phosphate (1 mg/ml) in substrate buffer (1 mol/l diethanolamine, 0.5 mmol/l MgCl_2_, pH 9.8) was used as substrate, and colour development was measured spectrophotometrically at 405 nm (Bio‐Rad, Tokyo, Japan) 30 min. later.

Cross‐reaction between antibodies against P14 and intact human α3(IV)NC1 was investigated using inhibition ELISA. In brief, polystyrene microtitre plates were coated with P14. The diluted sera were pre‐incubated with either soluble human α3(IV)NC1 from 0.1 to 10 mg/l or P14 from 0.1 to 10 μmol/l at 37°C for 60 min., respectively. The mixtures were then transferred to the P14‐coated microtitre plates, and the bound antibodies were detected with alkaline phosphatase‐conjugated secondary antibodies, as described above.

### Statistical analyses

Differences of quantitative parameters were assessed using the *t‐*test for data that were normally distributed, or nonparametric test for data that were not normally distributed. Differences of qualitative data were compared using chi‐square test and Fisher's exact test. Data from animal experiments are expressed as mean ± S.E.M. All statistical analyses were two‐tailed, and *P* value <0.05 was considered significant. Analysis was performed using SPSS statistical software package, version 13.0 (SPSS Inc., Chicago, IL, USA).

## Results

### Induction of anti‐GBM nephritis by immunization with linear peptides spanning Goodpasture antigen

Twenty‐four overlapping linear peptides were synthesized covering the whole sequence of human α3(IV)NC1 (Table 1).

**Table 1:** Sequences of each linear peptide on α3(IV)NC1 and T cell responses

Peptide	Sequence from N‐Terminus	Position from N‐terminus
P1	SPATWTTRGFVFTRHSQTTA	1–18
P2	VFTRHSQTTAIPSCPEGTVPL	9–29
P3	TAIPSCPEGTVPLYSGFSFLFV	17–38
P4	LYSGFSFLFVQGNQRAHGQD	29–48
P5	QGNQRAHGQDLGTLGSCLQR	39–58
P6	LGTLGSCLQRFTTMPFLFCN	49–68
P7	FTTMPFLFCNVNDVCNFASR	59–78
P8	VNDVCNFASRNDYSYWLSTPA	69–88
P9	NDYSYWLSIPALMPMNMAPI	79–98
P10	ALMPMNMAPITGRALEPYIS	89–108
P11	TGRALEPYISRCTVCEGPAI	99–118
P12	RCTVCEGPAIAIAVHSQTTD	109–128
P13	AIAVHSQTTDIPPCPHGWIS	119–138
P14	TDIPPCPHGWISLWKGFSFIMF	127–148
	LWKGFSFIMFTSAGSEGTGQ^a^	139–158
P15	TSAGSEGTGQALASPGSCLE	149–168
P16	ALASPGSCLEEFRASPFLEC	159–178
P17	EFRASPFLECHGRGTCNYYS	169–188
P18	HGRGTCNYYSNSYSFWLASL	179–198
P19	NSYSFWLASLNPERMFRKPI	189–208
P20	NPERMFRKPIPSTVKAGELE	199–218
P21	PSTVKAGELEKIISRCQVCM	209–228
P22	KIISRCQVCMKKRH	219–232
P23	KGFSFIMFTSAGSE	141–154
P24	FIMFTSAGSEGTGQ	145–158
P14a	TDIPPCPHGWISL	127–139
P14b	CPHGWISLWKGFS	132–144
P14c	ISLWKGFSFIMFT	137–149

a This sequence was not available due to synthetic difficulties. Two truncated peptides derived from it were then designed and successfully synthesized as P23 and P24.

**Figure 1:**
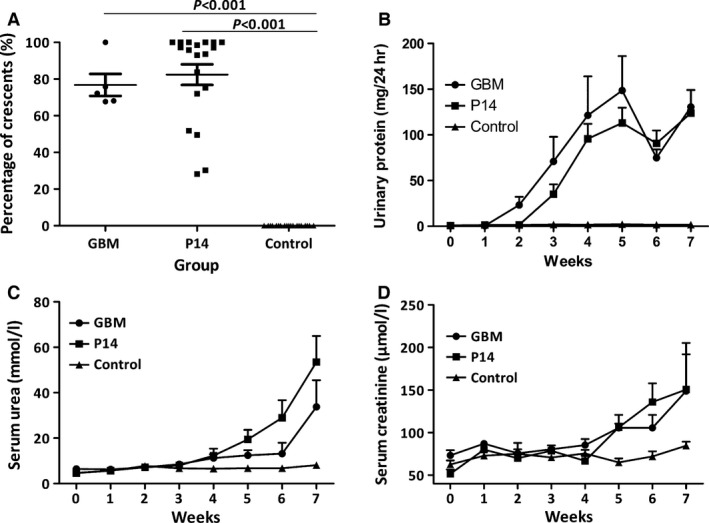
Clinical spectrum of WKY rats immunized with P14 (α3_127‐148_), bovine glomerular basement membrane (GBM, positive control) and complete Freund's adjuvant (CFA) (negative control). High percentages of cellular crescents were shown in the glomeruli of positive controls and P14 immunization rats, but not in negative controls (**A**). Rats that were immunized with P14 and GBM had significantly increased urinary protein compared to negative control group (**B**) and decreased renal function as measured by blood urea nitrogen (**C**) and serum creatinine (**D**).

Nineteen of 21 (90.5%) rats immunized with P14, α3_127‐148_, and developed severe crescentic glomerulonephritis (Fig. 1A). The animals showed proteinuria by week 3 after immunization (Fig. 1B). BUN and serum creatinine (Scr) increased by week 4 and 5, respectively (Fig. 1C and D). Then, the rats developed severe proteinuria (130.3 ± 17.6 *versus* 1.7 ± 0.3 mg/24 hrs, *P* < 0.001) and high levels of serum creatinine (152.5 ± 28.9 *versus* 84.3 ± 5.2 μmol/l, *P* = 0.031) and BUN (50.8 ± 14.1 *versus* 8.2 ± 0.6 mmol/l, *P* = 0.007) and were killed at week 7.

**Figure 2:**
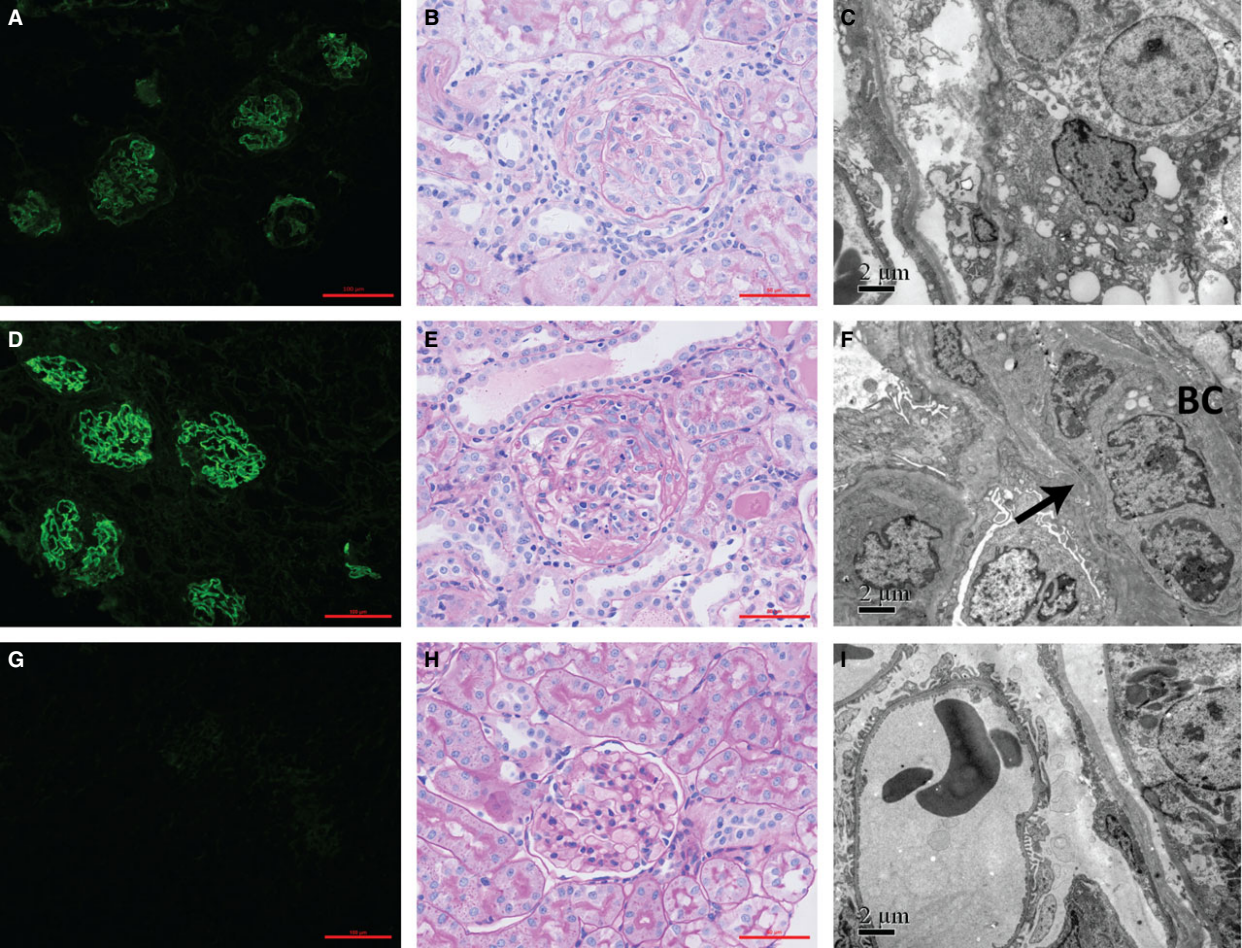
P14 immunization on WKY rats induced severe crescentic glomerulonephritis. Immunofluorescent staining of IgG was detectable along GBM in positive controls (**A**) and P14 immunization rats (**D**), but not in negative controls (**G**). Crescent formation was observed in positive controls (**B**) and P14 immunization rats (**E**), but not in negative controls (**H**). On electron microscope, the fracture and shrinking of GBM was identified in the glomeruli with crescent formation in positive controls (**C**) and P14 immunization group (**F**), without electron dense deposits. No GBM damage was observed on electron microscope in negative controls (**I**). BC: Bowman's capsule.

After killing, kidney injury was evaluated by direct immunofluorescence, light microscopy and electron microscopy. All the 19 rats immunized by P14 presented with linear deposits of IgG along GBM on direct immunofluorescence (Fig. 2D). The percentage of crescent formation in glomeruli was 82.4 ± 5.6%, with cellular crescents predominant (Fig. 2E). Lymphocyte and monocyte infiltration and tubular atrophy were found in the kidney interstitia accompanied by crescentic glomerulonephritis. On electron microscope, the fracture and shrinking of GBM was identified in the glomeruli with crescent formation, without electron dense deposits (Fig. 2F).

None of the rats immunized by other peptides developed any kidney injury. The urinary protein and the levels of serum creatinine and BUN were comparable to negative controls. No linear IgG staining or crescent formation was observed in the glomeruli.

All (6/6, 100%) the rats of positive controls developed severe crescentic glomerulonephritis as expected after the immunization with bovine GBM (Fig. 1A). All the rats showed proteinuria by week 2 (Fig. 1B). BUN and serum creatinine increased significantly and reached to 33.7 ± 21.7 mmol/l and 148.8 ± 48.8 μmol/l by the end of week 7 (Fig. 1C and D). On kidney histopathological examinations, linear staining of IgG along GBM was shown with 76.8 ± 6.0% of crescent formation in glomeruli (Fig. 2A–C). No (0/6, 0%) rat of negative controls had kidney injury after the immunization with complete Freund's adjuvant (CFA; Fig. 2G–I).

### Autoimmune disorder resulting from P14 immunization

#### Antibody production

**Figure 3:**
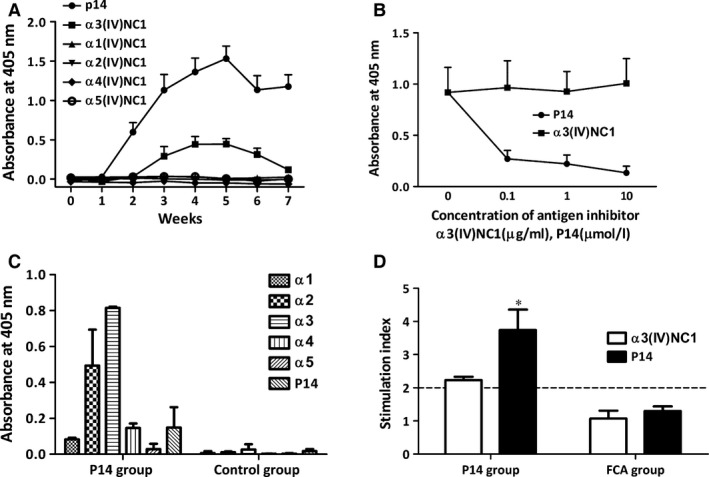
Immune responses to the P14 immunization on WKY rats. Serum antibodies from P14‐immunized rats recognized the immunogen peptide P14 and human α3(IV)NC1 protein (**A**). The binding between anti‐P14 antibodies and P14 was inhibited by P14 itself but not by α3(IV)NC1, using antigen inhibition ELISA (**B**). The elutes from P14‐immunization rat kidneys recognized P14, human α3(IV)NC1 and α2(IV)NC1 (**C**). Antigen‐specific lymphocyte proliferations towards P14 and α3(IV)NC1 were detectable in P14‐immunization rats, but not in negative controls (**D**). Cut‐off line (…) showed the level taken as representing positive response (SI = 2.0). *P = 0.014 compared with negative control.

Antigen specificity of the antibodies in sera and kidney elutes was detected by ELISA using P14 and recombinant human α1‐5(IV)NC1 as solid‐phase ligands (Fig. 3A). Rats immunized with P14 developed a robust serum antibody response to P14 itself by week 2 after immunization. To our surprise, 1 week later, the rats developed lower levels of antibodies towards intact human α3(IV)NC1. The responses to P14 and α3(IV)NC peaked at week 5 after immunization. Although the levels of antibodies decreased after week 5, the clinical manifestations of animals deteriorated progressively and rapidly and had to be killed by week 7. No serum reaction against other chains of human α(IV)NC1 was identified in the P14‐immunized rats.

Inhibition ELISA was performed to detect whether there was cross‐reaction between antibodies against P14 and intact human α3(IV)NC1 (Fig. 3B). The antibodies binding to P14 was strongly inhibited by soluble P14 but not by human α3(IV)NC1. The absence of cross‐reaction of antibodies against α3(IV)NC1 and P14 indicated that they were two distinct populations. Therefore, intramolecular epitope spreading occurred from linear epitopes on P14 to conformational epitopes on human α3(IV)NC1, during the disease progression of P14 induced EAG.

Antibodies were eluted from kidneys of two rats immunized with P14 and two rats with CFA immunization, respectively. Kidney elutes from P14 immunization rats demonstrated high levels of IgG binding to human α3(IV)NC1 but low‐level binding to the immunogen P14, in contrast to the high level anti‐P14 antibodies in the circulation (Fig. 3C). A moderate binding to α2(IV)NC1 was also found from the kidney elutes, while only mild or no binding to α1, α4 or α5(IV)NC1 was observed in the kidney elutes.

Two of the rats in the P14 immunization group developed antibody response to P14 but not to α3(IV)NC1, and no glomerulonephritis was observed in these rats.

#### Lymphocyte proliferation

Lymphocyte response to P14 and intact α3(IV)NC1 were tested in nine rats immunized with P14 by proliferation assay using ^3^H‐thymidine. Lymphocytes from draining lymph nodes showed significant T cell proliferative responses to P14 (SI 3.7 ± 0.6 *versus* 1.3 ± 0.1, *P* = 0.014) and α3(IV)NC1 (SI 2.2 ± 0.1 *versus* 1.1 ± 0.2, *P* = 0.091) compared to five rats of negative controls (Fig. 3D).

### The core immunogenic region on P14 for inducing crescentic glomerulonephritis

P14 was truncated into three 13‐mer peptides overlapping by eight amino acids, designated as P14a (α3_127‐139_), P14b (α3_132‐144_) and P14c (α3_137‐149_) to investigate the core immunogenic region on P14.

**Figure 4:**
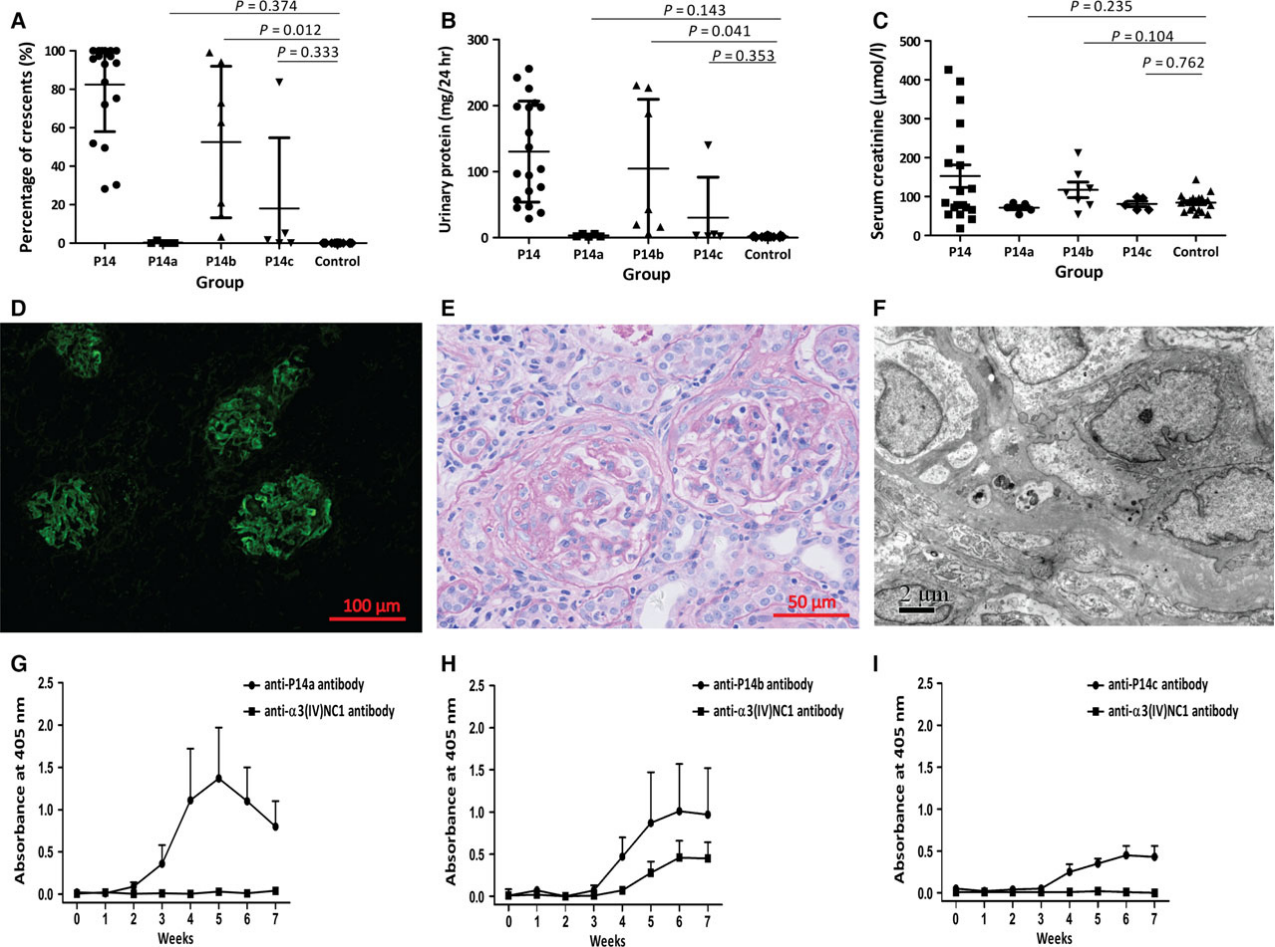
The core immunogenic region on P14. P14 was truncated into three 13‐mer peptides designated as P14a, P14b and P14c. High percentage of crescents were shown in the kidney tissues of rats immunized with P14b, but not with P14a or P14c (**A**). Urinary protein (**B**) was significantly increased in the rats immunized with P14b, but not with P14a or P14c. Serum creatinine (**C**) was also increased in the rats immunized with P14b, but not with P14a or P14c. The rats immunized with P14b showed linear IgG deposits along GBM on direct immunofluorescence (**D**), crescent formation on light microscope (**E**) and no electron dense deposit on electron microscope (**F**). The rats immunized with P14b developed antibodies against P14b itself as well as antibodies against the intact human α3(IV)NC1 (**H**). The rats immunized with P14a or P14c only produced antibodies recognizing the immunogens themselves (**G** and **I**).

The rats immunized with P14b developed elevated levels of urinary protein (104.5 ± 39.7 *versus* 1.7 ± 0.3 mg/24 hrs, *P* = 0.041) and serum creatinine (117.4 ± 20.0 *versus* 84.3 ± 5.2, *P* = 0.104) compared to negative controls (Fig. 4B and C). Kidney tissues showed linear deposits of IgG along GBM (Fig. 4D) and high percentage of crescent formation (52.6 ± 14.9% *versus* 0.0 ± 0.0%, *P* = 0.012; Fig. 4A,E and F). The rats immunized with P14c showed slightly elevated levels of urinary protein (30.4 ± 27.3 *versus* 1.7 ± 0.3 mg/24 hrs, *P* = 0.353; Fig. 4B) and crescent formation (18.1 ± 16.4% *versus* 0.0 ± 0.0%, *P* = 0.333; Fig. 4A), but the differences were not statistical significant compared to negative controls. No rats immunized with P14a developed any kidney injury.

All rats produced antibodies targeting the immunogens, but only the rats immunized by P14b developed antibodies towards the intact human α3(IV)NC1 (Fig. 4H), while the rats immunized by P14a or P14c did not (Fig. 4G and I).

### Identification of critical amino acid residues on P14

To determine critical motif from the 22 amino acid residues on P14, each amino acid of P14 was substituted by alanine in a sequential order and 21 new peptides were resynthesized after substitution (Table 2). The peptide with lysine_141_ substituted by alanine was failed for synthesis because of its strong hydrophobicity.

**Table 2:** Sequential residue substitution at each position of P14

Residue of substitution	Sequence from N‐terminus	Position from N‐terminus
P14	TDIPPCPHGWISLWKGFSFIMF	127–148
T	***A***DIPPCPHGWISLWKGFSFIMF	127
D	T***A***IPPCPHGWISLWKGFSFIMF	128
I	TD***A***PPCPHGWISLWKGFSFIMF	129
P	TDI***A***PCPHGWISLWKGFSFIMF	130
P	TDIP***A***CPHGWISLWKGFSFIMF	131
C	TDIPP***A***PHGWISLWKGFSFIMF	132
P	TDIPPC***A***HGWISLWKGFSFIMF	133
H	TDIPPCP***A***GWISLWKGFSFIMF	134
G	TDIPPCPH***A***WISLWKGFSFIMF	135
W	TDIPPCPHG***A***ISLWKGFSFIMF	136
I	TDIPPCPHGW***A***SLWKGFSFIMF	137
S	TDIPPCPHGWI***A***LWKGFSFIMF	138
L	TDIPPCPHGWIS***A***WKGFSFIMF	139
W	TDIPPCPHGWISL***A***KGFSFIMF	140
K	TDIPPCPHGWISLW**A**GFSFIMF^a^	141
G	TDIPPCPHGWISLWK***A***FSFIMF	142
F	TDIPPCPHGWISLWKG***A***SFIMF	143
S	TDIPPCPHGWISLWKGF***A***FIMF	144
F	TDIPPCPHGWISLWKGFS***A***IMF	145
I	TDIPPCPHGWISLWKGFSF***A***MF	146
M	TDIPPCPHGWISLWKGFSFI***A***F	147
F	TDIPPCPHGWISLWKGFSFIM***A***	148

a This sequence was not available for peptide synthesis due to hydrophobicity.

Twenty‐one groups (five rats per group) of rats were immunized with the series of amino acid mutation peptides. EAG was evaluated in each group. Critical amino acid was identified by the loss of nephritogenic function after substitution, resulting neither crescent formation in kidney nor clinical manifestations of proteinuria or rise of serum creatinine or BUN.

**Figure 5:**
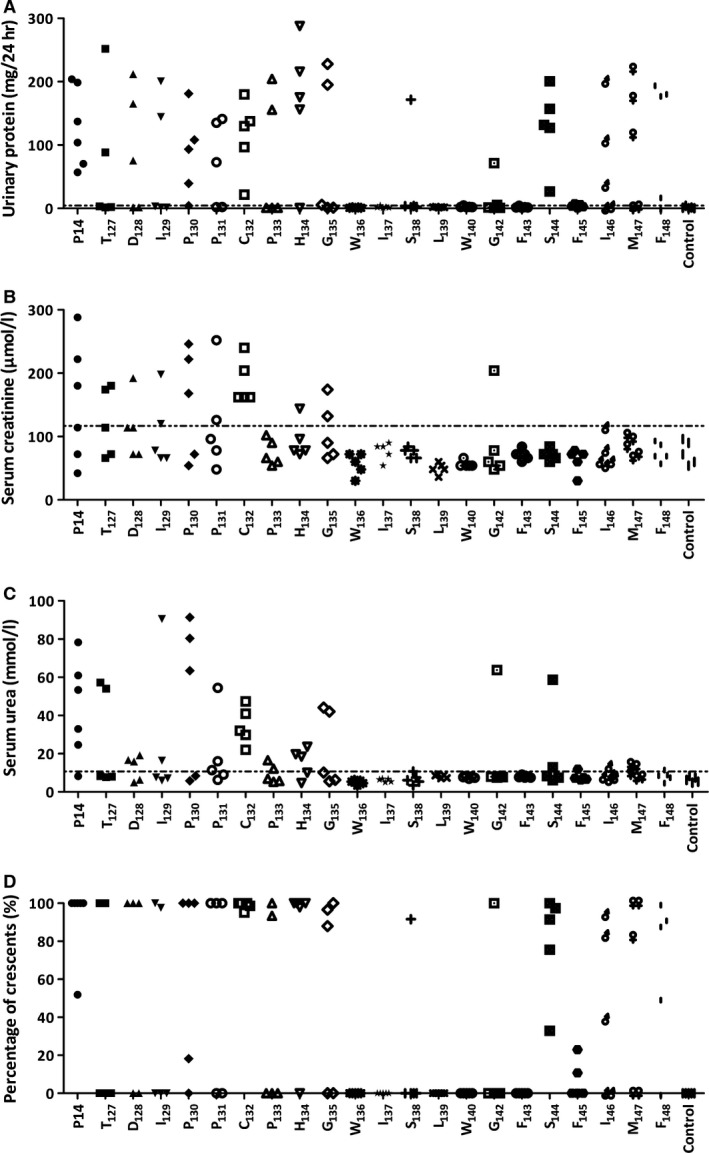
Defining the critical residues on P14 peptide. WKY rats were immunized with alanine substituted 22‐mers, with each peptide having one residue substitution in sequential order. Tryptophan_136_, isoleucine_137_, leucine_139_, tryptophan_140_ and phenylalanine_143_ replaced peptides failed to induce EAG with proteinuria (**A**), elevated serum creatinine (**B**) or urea (**C**) or crescentic glomerulonephritis (**D**). The line (…) is the normal range from negative controls.

We found that when tryptophan (W)_136_, isoleucine (I)_137_, leucine (L)_139_, tryptophan (W)_140_ or phenylalanine (F)_143_ were substituted by alanine, the mutated P14 lost its nephritogenicity. No rat developed EAG after the immunization with P14 possessing any of the above amino acid mutations (Fig. 5, Table S1). The urinary protein excretion (1.9 ± 0.2 mg/24 hrs), serum creatinine concentration (61.9 ± 3.0 μmol/l) and BUN (7.0 ± 0.3 mmol/l) were all in normal range in the animals immunized with the five above‐mentioned mutated peptides. No rat developed crescent in the glomeruli, nor IgG staining on the kidney.

P14 with other amino acid substitutions could induce EAG in at least one rat of its group.

### Epitope spreading and kidney injury

**Figure 6:**
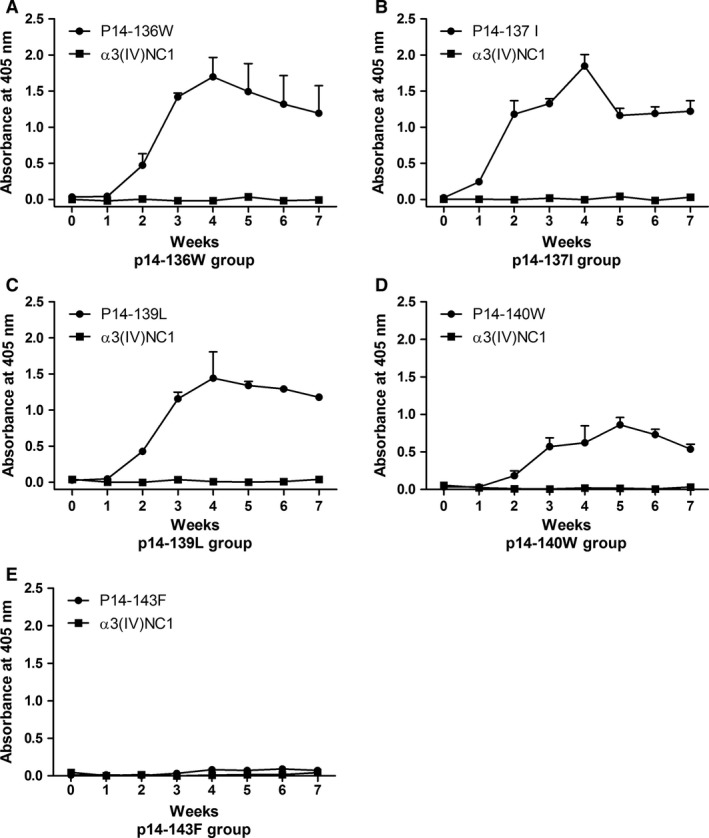
Serum antibodies from the rats immunized with sequential amino acid substituted peptides. Serum antibody recognized the immunogen itself but not intact α3(IV)NC1 in the rats immunized with tryptophan_136_, isoleucine_137_, leucine_139_ or tryptophan_140_ substitution peptide of P14 (**A–D**). No antibody was found towards the immunogen or α3(IV)NC1 in the rats immunized with phenylalanine_143_ substitution peptide of P14 (**E**).

The associations between epitope spreading to α3(IV)NC1 and the severity of kidney injury were further analysed. Serum antibody productions were detected for all groups. We found that the rats immunized with P14 having amino acid substitutions of W_136_, I_137_, L_139_ or W_140_ developed antibodies against the immunogen itself, but no antibody towards the intact α3(IV)NC1. The rats immunized with F_143_ mutation peptide produced no serological reaction to either the immunogen or α3(IV)NC1 (Fig. 6). In other groups of rats which developed crescentic glomerulonephritis after the immunization of mutated peptides, both anti‐immunogen and anti‐α3(IV)NC1 antibodies were produced.

**Figure 7:**
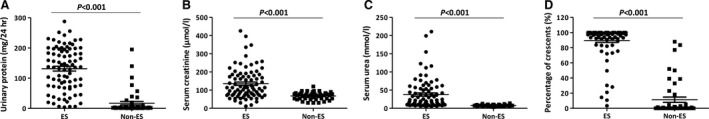
Comparison of anti‐GBM nephritis severity on WKY rats immunized with P14 and its mutations, between those possessing anti‐α3(IV)NC1 antibodies and those not. The urinary protein excretion (A), serum creatinine concentration (B), blood urea nitrogen level (C) and the percentage of crescent formation (D) in glomeruli were all significantly higher in the rats with antibodies against both the immunogen and α3(IV)NC1, compared with those with anti‐immunogen antibodies alone ES: epitope spreading occurred from the immunogen to α3(IV)NC1. Non‐ES: epitope spreading not occurred with anti‐immunogen antibody alone.

The percentage of crescent formation in glomeruli (90.5 ± 2.6 *versus* 11.3 ± 3.5%, *P* < 0.001), urinary protein excretion (131.5 ± 7.9 *versus* 17.3 ± 5.9 mg/24 hrs, *P* < 0.001), serum creatinine concentration (136.2 ± 9.1 *versus* 67.8 ± 3.0 μmol/l, *P* < 0.001) and BUN (39.5 ± 4.7 *versus* 8.1 ± 0.3 mmol/l, *P* < 0.001) were all significantly higher in the rats with antibodies against both the immunogen and α3(IV)NC1, compared with those with anti‐immunogen antibodies alone (Fig. 7). Thus, the amino acid residues of W_136_, I_137_, L_139_ and W_140_ were identified as critical motif for the pathogenicity of P14, probably through the process of epitope spreading to α3(IV)NC1 after immunization. F_143_ might be more important for the immunogenicity of P14.

## Discussion

In the present study, we identified that one T cell epitope P14, α3_127‐148_, could successfully induce crescentic glomerulonephritis and autoimmunity towards α3(IV)NC1 after the immunization on WKY rats. The autoimmune disorders, clinical spectrum and kidney pathological features were in accordance to human Goodpasture disease. It is intriguing that one single immunization of the synthetic peptide could be sufficient to induce such a fulminant autoimmune disease. To our knowledge, it is the first time that one linear peptide within human α3(IV)NC1 was proven to induce anti‐GBM nephritis in WKY rats, which indicates that P14 contains nephritogenic T cell epitope(s). To some extent, these findings gave indirect evidence that P14 may also be nephritogenic for human Goodpasture disease. These findings will help us to understand the aetiology of Goodpasture disease, and more importantly to be useful for the potential development of specific immunotherapies based on the critical amino acid motif discovered in the current study.

P14, α3_127‐148_, is a special region on Goodpasture antigen. Its unique features might render its capacity to induce EAG: firstly, P14 is highly scissile to protease cathepsin D and E and could be easily destroyed in early antigen‐presenting process under normal circumstances. Thus, P14 is a ‘cryptic epitope’ to human immune system 36, 37. Secondly, P14 has a high binding affinity to HLA‐DRB1*1501, a major susceptible allele for Goodpasture disease onset, which may be advantageous to the activation of P14‐specific T cells once it is presented by antigen‐presenting cells 31, 38, 39. Thirdly, P14 contains the primary sequence of E_B_, which is one of the two classical conformational epitopes recognized by anti‐GBM antibodies. Our previous study in anti‐GBM patients indicated that antibodies against E_B_ were an independent risk factor for renal failure 40.

In the current study, we found that P14 not only induced severe EAG but also initiated intramolecular epitope spreading, resulting in circulating antibodies against intact human α3(IV)NC1 after the production of anti‐P14 antibodies. These two kinds of antibodies had no cross‐reaction, which indicated that antibodies against epitopes external to P14 on α3(IV)NC1 evolved during disease progression. Moreover, the kidney elutes also confirmed the deposition of antibodies against α3(IV)NC1 and α2(IV)NC1 to a lesser extent. Epitope spreading was reported in other autoimmune animal models, including systemic lupus erythematosus, encephalomyelitis, multiple sclerosis and thyroiditis 31, 41, 42, 43. Bolten *et al*. first described intramolecular B cell epitope spreading after the rats were immunized with a 13 amino acid T cell epitope 44. In the present study, we proved that the T cell epitope P14 on α3(IV)NC1 could induce B cell epitope spreading. Our previous study found that antibodies against P14 existed in the circulation of patients with anti‐GBM disease. Therefore, P14 is the mutual epitope for both T cells and B cells. It was reasonable to deduce that P14 is the initial epitope for human Goodpasture disease, which induces diversified antibody responses external to itself.

We previously reported the observations that anti‐GBM patients with more aggravated renal dysfunction had broader antigen spectrum compared to those with normal renal function 19. In the present study, we found that all the 21 rats immunized with P14 developed antibody response to itself, but two of them did not develop antibodies to α3(IV)NC1 and no kidney injury occurred. In all groups, proteinuria, serum creatinine, BUN and percentage of crescents in glomeruli were significantly higher in the rats with epitope spreading than those without. These findings imply that epitope spreading is a possible causal step, which provokes the burst of the disease. An alternative explanation might be that P14 itself could fold into a three‐dimensional structure, which was nephritogenic‐like E_B_. Further investigations are needed to elucidate it.

We further characterized the critical amino acid residues on P14 using amino acid substitution. The mutated P14 lost its pathogenic capacity with each substitution of W_136_, I_137_, L_139_ or W_140_, failed to induce kidney disease or epitope spreading, but maintained the immunogenic character. These four amino acids were proposed to be the critical amino acid motif on P14. Using HLA‐DRB1*1501 transgenic mice, Ooi *et al*. found that T cell activity was lost by substitution of V_137_, W_140_, G_142_ or F_143_ 31. Goodpasture antigen is a highly conservative protein during evolution, with overall 91% homology on amino acid sequence between human and rodent 45. V137 is corresponding to I137 in human α3(IV)NC1). I_137_ and W_140_ were found critical to pathogenicity in our present study. G_142_ and F_143_ were critical for B cell recognition in Goodpasture patients as revealed in our previous study 35. Tryptophan (W), isoleucine (I), leucine (L) and phenylalanine (F) were all hydrophobic amino acids, which were buried within α3(IV)NC1 and normally inaccessible for antibody binding 46. The bonds of W_136_‐I_137_ and L_139_‐W_140_ were sensible to protease and could easily be destroyed in antigen processing, which renders it as a ‘cryptic’ T cell epitope 37. These mechanisms might explain why these amino acid residues were critical for inducing autoimmune response, but further studies are still needed.

P14 with amino acid substitution for F_143_ also failed to induce the disease by the loss of immunogenic character. We further truncated P14 into a 13‐mer peptide including F_143_, which was easier to be presented by MHC molecules. Then, the peptide with substitution of F_143_ maintained its pathogenic and immunogenic characters (data not shown); thus, F_143_ was excluded to be one of the critical residues for pathogenicity.

In conclusion, we identified a nephritogenic T cell epitope on human Goodpasture antigen, designated as P14 (α3_127‐148_), which not only induced severe anti‐GBM nephritis but also initiated epitope spreading in WKY rats. The critical amino acid motif was W_136_I_137_xL_139_W_140_, which were hydrophobic and sensible to protease.

## Disclosure

All the authors declared no competing interests.

## Supporting information


**Table S1.** Clinical spectrum of rats immunized with substituted peptides derived from P14.
