# Supplementary material for: The pathogenicity of T cell epitopes on human Goodpasture antigen and its critical amino acid motif
Source: J Cell Mol Med. 2017 Mar 10;21(9):2117–28. doi: 10.1111/jcmm.13134 (PMC5571546; doi:10.1111/jcmm.13134)
Supplement: Supplementary file 1 — Table S1. Clinical spectrum of rats immunized with substituted peptides derived from P14. [file JCMM-21-2117-s001.docx]

**Supplementary table 1. Clinical spectrum of rats immunized with substituted peptides derived from P14.**

| Residue of substitution | Sequence from N-terminus | Position from N-terminus | Urinary protein (mg/24h) | Serum creatinine (μmol/L) | Percentage of crescents (%) |
| --- | --- | --- | --- | --- | --- |
| P14 | TDIPPCPHGWISLWKGFSFIMF | 127-148 | 203.9 | 288.0 | 100 |
| T | ***A***DIPPCPHGWISLWKGFSFIMF | 127 | 251.8 | 180.0 | 100 |
| D | T***A***IPPCPHGWISLWKGFSFIMF | 128 | 211.8 | 192.0 | 100 |
| I | TD***A***PPCPHGWISLWKGFSFIMF | 129 | 200.5 | 198.0 | 100 |
| P | TDI***A***PCPHGWISLWKGFSFIMF | 130 | 181.1 | 246.0 | 100 |
| P | TDIP***A***CPHGWISLWKGFSFIMF | 131 | 141.0 | 252.0 | 100 |
| C | TDIPP***A***PHGWISLWKGFSFIMF | 132 | 180.0 | 240.0 | 100 |
| P | TDIPPC***A***HGWISLWKGFSFIMF | 133 | 204.3 | 102.0 | 100 |
| H | TDIPPCP***A***GWISLWKGFSFIMF | 134 | 287.8 | 144.0 | 100 |
| G | TDIPPCPH***A***WISLWKGFSFIMF | 135 | 227.8 | 174.0 | 100 |
| W | TDIPPCPHG***A***ISLWKGFSFIMF | 136 | 1.65 | 72.0 | 0 |
| I | TDIPPCPHGW***A***SLWKGFSFIMF | 137 | 4.51 | 90.0 | 0 |
| S | TDIPPCPHGWI***A***LWKGFSFIMF | 138 | 171.5 | 84.0 | 91.7 |
| L | TDIPPCPHGWIS***A***WKGFSFIMF | 139 | 2.6 | 60.0 | 0 |
| W | TDIPPCPHGWISL***A***KGFSFIMF | 140 | 4.6 | 66.0 | 0 |
| *K* | *TDIPPCPHGWISLW****A****GFSFIMF^a^* | *141* | *-* | *-* | *-* |
| G | TDIPPCPHGWISLWK***A***FSFIMF | 142 | 71.3 | 204.0 | 100 |
| F | TDIPPCPHGWISLWKG***A***SFIMF | 143 | 4.2 | 84.0 | 0 |
| S | TDIPPCPHGWISLWKGF***A***FIMF | 144 | 200.6 | 84.0 | 100 |
| F | TDIPPCPHGWISLWKGFS***A***IMF | 145 | 7.4 | 78.0 | 22.9 |
| I | TDIPPCPHGWISLWKGFSF***A***MF | 146 | 200.6 | 114.0 | 94.0 |
| M | TDIPPCPHGWISLWKGFSFI***A***F | 147 | 219.8 | 102.0 | 100 |
| F | TDIPPCPHGWISLWKGFSFIM***A*** | 148 | 196.7 | 96.0 | 100 |

*^a^* This sequence was not available for peptide synthesis due to hydrophobicity.
